# Incidence and Influencing Factors of New Hepatitis B Infections and Spontaneous Clearance: A Large-Scale, Community-Based Study in China

**DOI:** 10.3389/fmed.2021.717667

**Published:** 2021-11-18

**Authors:** Haiyang Hu, Yangfan Shen, Ming Hu, Yang Zheng, Kaijin Xu, Lanjuan Li

**Affiliations:** State Key Laboratory for Diagnosis and Treatment of Infectious Diseases, Collaborative Innovation Center for Diagnosis and Treatment of Infectious Diseases, School of Medicine, The First Affiliated Hospital, Zhejiang University, Hangzhou, China

**Keywords:** hepatitis B surface antigen, hepatitis B virus, natural history, incidence rate, influencing factor

## Abstract

**Background:** Hepatitis B surface antigen (HBsAg) is widely used in hepatitis B screening, and HBsAg seroclearance indicates hepatitis B eradication. Few studies have explored the incidence of and determinants for spontaneous seroclearance using a long-term follow-up cohort study. Our research aimed to examine the incidence of and influencing factors for hepatitis B virus infection and spontaneous clearance of HBsAg from a large-scale cohort in China.

**Methods:** A total of 151,926 resident individuals in Tongxiang underwent HBsAg screening at least thrice in a 7-year period. Serum samples collected at baseline and follow-up examinations were tested for HBsAg. Cox proportional hazard models were used to analyze determinants of HBsAg seroclearance and persistent HBsAg presence.

**Results:** Among the 151,926 participants, new hepatitis B infections occurred in 4,497 participants, yielding an incidence rate of 571.38 per 100,000 person-years. The incidence rate for males was higher than that for females. In the multivariate Cox regression analysis, female gender, alcohol drinking history, hepatitis family history and middle-age group were predictors for persistent positive HBsAg status.

**Conclusions:** The incidence rate of new hepatitis B infections was 571.38 per 100,000 person-years. Male and aged people in this community cohort have a higher infection rate. Alcohol drinking and hepatitis family history were risk factor leading to chronic infection. Female and middle-aged people were prone to persistent positive HBsAg status.

## Introduction

Hepatitis B is an infectious disease induced by hepatitis B virus (HBV) that mainly invades the liver and can induce a variety of liver diseases, such as acute or chronic hepatitis, hepatic failure, liver cirrhosis and hepatic carcinoma (1, 2). In 2015, the WHO estimated that 257 million persons (3.5% of the global population) were living with chronic HBV infection (3). Most of them were adults who had no hepatitis B vaccine immunization in infancy. These individuals suffer from HBV infection and are also the source of infection for other non-vaccinated people. After infection with HBV, 1–2% of cases evolve into fulminant hepatic failure, and 5–10% of adult cases evolve into chronic infection (1). In addition, others typically have no symptoms and progress to spontaneous clearance of HBV within ~3 months (4–6). Since its discovery in the 1960s, hepatitis B surface antigen (HBsAg, also commonly known as Australia antigen) has become an important serological marker for screening for HBV infection (7). HBsAg is a distinctive surface antigen of HBV with an envelope protein and excess coat particles. HBsAg is positive in serological testing in acute and chronic hepatitis B infections (8) and indicates a current HBV infection. For most people, HBsAg spontaneously vanishes via seroconversion to anti-HBs antibodies (an antibody for the hepatitis B surface antigen) in a few months, indicating viral clearance. For cases evolving into chronic HBV infection, HBsAg remains positive with other serologic marker alterations. Individuals with chronic HBV infection may not develop clinical symptoms for as long as 30 years before apparent hepatic impairment, and a patient will not be aware of his or her disease (9). Therefore, for early diagnosis and early intervention, HBV serological marker screening remains important in regions with a high prevalence of HBV infection for early diagnosis and early intervention (1, 2, 10).

In China, the prevalence of HBV infection was estimated to be 9.75% in 1992, as determined by HBsAg testing. Due to the Expanded Program on Immunization (EPI) for infants established in 1992, the prevalence declined to 7.18% in 2006, according to a national epidemiological survey (11, 12). Previous studies have reported the incidence rate of spontaneous HBsAg seroclearance in chronic infections to be between 0.5 and 1.4% annually. Statistical indicators including but not limited to increasing age, male sex, HBV genotype B, and low initial HBV-DNA levels indicated high rate of spontaneous HBsAg seroclearance in these cohort studies (13–17). However, long-term, community-based studies of a large cohort of HBV carriers to examine the full range of factors leading to HBsAg seroclearance are still lacking. Since 2010, we have conducted a population-based infectious disease cohort study in Zhejiang Province, China. This research is based on a Demonstration Area Construction project aimed at whole-population infectious disease screening, intervention and follow-up, and it has enrolled over 200 thousand participants in the county of Tongxiang, representing approximately one-quarter of the local population (815.8 thousand), for HBsAg screening. In the following 7 years, all participants were invited to receive a free HBsAg examination every year. All the adult members in this cohort have no HBV vaccination history during the neonatal period and underwent no additional HBV vaccine administration. Therefore, this study provides an incidence rate of HBV infection and elucidates the factors influencing spontaneous HBsAg seroclearance in an adult population based on a remarkably large-scale, prospective cohort data set.

## Methods

### Study Cohort

This research began in October 2010 and ended in December 2020. The study cohort was enrolled before October 2010 in the county of Tongxiang, Zhejiang Province, China. All cohort participants born after 1992 received routine administration of HBV vaccination during infancy and then underwent no additional HBV vaccine administration. The baseline survey was conducted with the help of the Mega-Project for National Science and Technology Development for the “13th Five-Year Plan of China” and the Health Commission of Zhejiang Province. After receiving appropriate training by the lead researchers, the physicians of each participating hospital began to conduct medical examinations, interviews, and laboratory tests on subjects who volunteered for free medical and health examinations. Approximately 700 physicians from 50 hospitals in Tongxiang were invited by the Department of Health of Zhejiang Province to participate.

**Figure 1:**
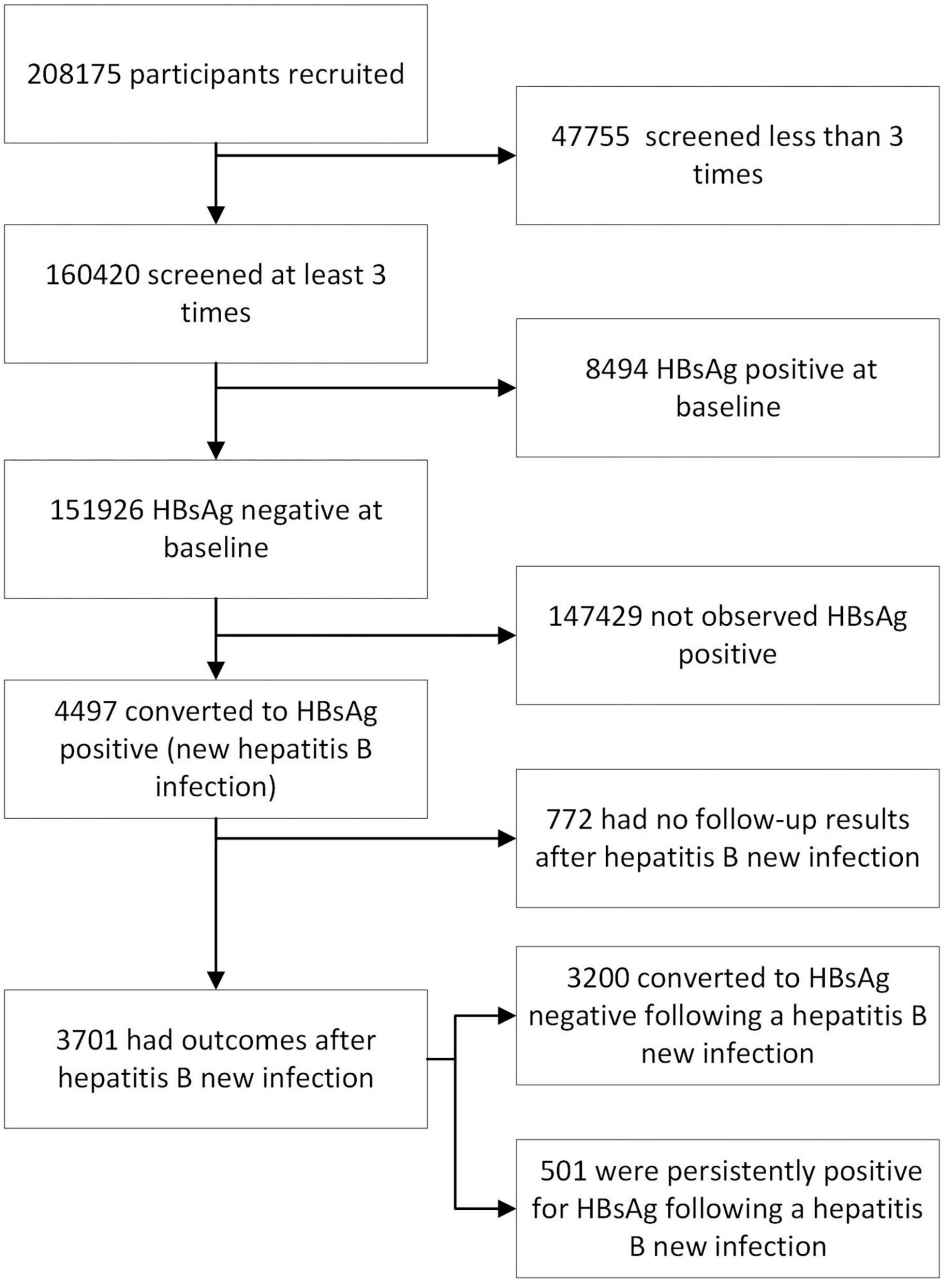
The flowchart for this cohort study (*n* = 208,175) of HBsAg status from 2010 to 2016 in Zhejiang Province, China.

In total, 208,175 residents in Tongxiang, representing approximately one-quarter of the whole resident population (815.8 thousand), voluntarily received a free HBsAg baseline survey before October 2010. Individuals who had lived in the county for more than 24 months were invited to participate in the study cohort. We invited every participant to receive an annual health examination in the local hospitals. During each visit, medical staff working for our research team inquired about symptoms and performed a physical examination accompanied by HBsAg testing for every participant. A total of 151,926 participants were HBsAg negative at the baseline survey and received at least 3 HBsAg screenings during the research period (Figure 1).

All cohort participants provided informed written consent, and informed written consent was obtained from guardians on behalf of minors. The study was approved by the Ethics Committee of The First Affiliated Hospital at the School of Medicine of Zhejiang University. All data were analyzed anonymously. The information collected consisted of basal demographics (sex, age, education level, marital status, medical insurance, family history, smoking history and alcohol drinking history) and laboratory tests (HBsAg).

### Serological Testing

A 5-mL venous blood sample was collected during the annual health examination and kept in a low temperature container (controlled from 4 to 10 μC) and delivered to Adicon Clinical Laboratories (Hangzhou, China) on the same day for sample processing and serological testing. Commercially available enzyme immunoassay kits (Acon Biotech Co., Hangzhou, China) were used for the HBsAg measurements. Verification of elevated test results was performed by retesting the samples using the same kits. Only samples that were positive on both tests were considered true positives. For the purpose of analysis, HBsAg positivity was considered indicative of current hepatitis B infection.

### Statistical Analysis

The person-years of follow-up was calculated from the date of enrolment to the date of the last blood test and the onset of the specific outcomes of interest. The time from HBV infection to HBsAg seroclearance was artificially defined as the midpoint of two adjacently continuous tests with different results (18). Participants without HBV infection or HBsAg seroclearance were defined as censored for specific study outcomes. Data were managed and analyzed using SPSS software version 25.0 (SPSS, Inc., Chicago, IL, USA). Categorical data, such as incidence rates of different sexes or age groups, were compared using the chi-square test, and *p*-values were adopted for pairwise comparisons. The Kaplan–Meier method was used to examine the cumulative probability of HBV infection, HBsAg seroclearance, and annual incidence rate, and univariate analysis of each variable was also performed. Annual incidence rates were calculated directly from the survival table, and a chi-square trend test was then performed to evaluate the pattern of change. The results of different levels of each variable were compared with log-rank tests. Cox proportional hazards models were used to analyze both the univariate and multivariate-adjusted rate ratios (with 95% confidence intervals) of HBsAg seroclearance/persistent positive HBsAg associated with various determinants. Variables significant in the univariate analyses were included in multivariate analyses. Statistical significance was determined by 2-tailed tests (*p* < 0.05).

## Results

### Basic Characteristics of Participants

We analyzed a total of 151,926 participants with more than 3 consecutive HBsAg tests. In total, 57,089 of participants were male, and 94,837 were female. The average age was 66.59 ± 11.68 years with a minimum age of 9 years and a maximum age of 106 years. Among all the participants, 4,497 were found to experience a HBsAg positive conversion (as shown in Table 1), indicating a new HBV infection. Another 147,429 participants were consistently observed to be HBsAg negative.

**Table 1:** Demographic characteristics of the 151,926 participants who were HBsAg negative at baseline and who experienced HBsAg positive conversion.

**Variables**	**HBsAg positive conversion**	**Total**	**Person-years of follow-up**	**Annual incidence rate (per 100,000)**	***p***
Gender					<0.001
Female	2,647	92,190	5.35	536.68	
Male	1,850	55,239	4.89	684.88	
Age group					<0.001
0–10	0	1	1.25	0.00	NA
11–20	6	60	3.92	2550.70	NA
21–30	13	561	3.57	648.38	a,b,c
31–40	52	2,722	4.45	428.90	a,b
41–50	199	10,878	4.78	382.51	a
51–60	527	24,778	5.15	413.06	a,b
61–70	1,825	56,658	5.46	590.17	c,d
71–80	1,264	38,369	5.29	622.61	c,d
81–90	561	16,384	4.59	745.76	c,d
91 and above	50	1,515	3.69	895.14	b,d
Total	4,497	151,926	5.18	571.38	

*a, b, c, d: each letter denotes a subset of age group categories whose incidence rates do not differ significantly from each other. The age groups of 0–10 and 11–20 years were not applicable in the statistics due to the insufficient sample size*.

### Incidence of New Hepatitis B Infection

**Figure 2:**
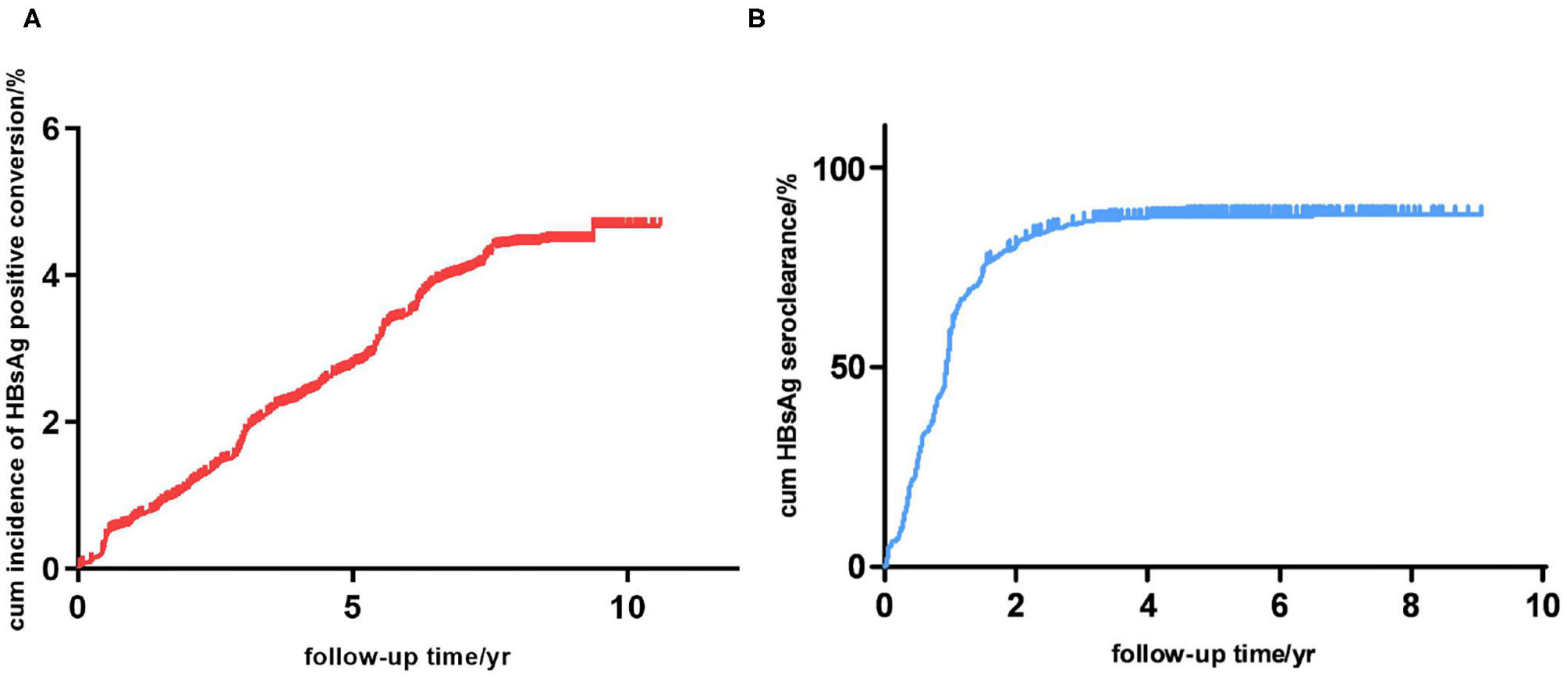
**(A)** Cumulative incidence of new hepatitis B infection in this study cohort (*n* = 151,926). **(B)** Cumulative incidence of HBsAg seroclearance in participants with hepatitis B infection (*n* = 4,497).

After 5.18 person-years of follow-up, new hepatitis B infections occurred in 4,497 participants, yielding an incidence rate of 571.38 per 100,000 person-years in average. The incidence rate of new hepatitis B infection for males was significantly higher than that for females (684.88 vs. 536.68 per 100,000 person-years, respectively, *p* < 0.001), and different age groups also had different incidence rates (*p* < 0.001). Incidence of new hepatitis B infection in 30–60 years age groups are comparatively lower than that of 20–30 and aged (60 years and above) groups. The new infection rate increased distinctly with age in the 60 years and above groups. The 11–20 years age group have an extremely high new infection incidence. They were not took into the saliency analysis due to the insufficient sample size. The cumulative incidence of new hepatitis B infections by 3, 5, and 10 years was 0.018, 0.028 and 0.047, respectively (Figure 2A). The annual new infection incidence rate exhibited a decreasing tendency (Supplementary Table 1). Overall infection rate of this cohort tend to a platform of 5 percent.

### Incidence and Influencing Factors of New Hepatitis B Infection Outcomes

A total of 4,497 participants were observed to have new hepatitis B infections; however, 796 of these participants were excluded from further study because of missing data. Among the remaining 3,701 participants, after 5.40 ± 1.85 person-years of follow-up (1.41 years after HBV infection), 3,200 (86.46%) were found to have a HBsAg seroclearance, while the other 501 (13.54%) were persistently HBsAg positive (Table 2). The cumulative incidence of HBsAg seroclearance at 1, 2, and 5 years was 0.573, 0.802 and 0.861, respectively (Figure 2B). Seroclearance was normally completed in the first 3 years, after which it reached a plateau. The annual incidence rate of HBsAg seroclearance was significantly different between the first 2 years and the following years (Supplementary Table 2). Noteworthy, spontaneous HBsAg seroclearance hardly occurred since the 5th year after infection. Overall seroclearance rate of this cohort tend to a platform of 86 percent.

**Table 2:** Outcomes and univariate analysis of HBsAg positive conversion.

**Variables**	**Negative conversion (1)**	**Persistent infection (2)**	**Total number**	***p***
Total	3,200 (86.46%)	501 (13.54%)	3,701	
**Gender**				<0.001
Male	1,384 (88.32%)	183 (11.68%)	1,567	
Female	1,816 (85.10%)	318 (14.90%)	2,134	
**Age group**				<0.001
11–20	0	0	0	
21–30	8 (80.00%)	2 (20.00%)	10	
31–40	26 (66.67%)	13 (33.33%)	39	
41–50	98 (65.77%)	51 (34.23%)	149	
51–60	227 (69.85%)	98 (30.15%)	325	
61–70	1,341 (87.36%)	194 (12.64%)	1,535	
71–80	1,033 (90.93%)	103 (9.07%)	1,136	
81–90	435 (92.16%)	37 (7.84%)	472	
91 and above	32 (91.43%)	3 (8.57%)	35	
**Hepatitis B family history**	Unavailable for 5 (0.14%) participants			<0.001
0	2,840 (88.09%)	384 (11.91%)	3,224	
1	358 (75.85%)	114 (24.15%)	472	
**Smoking history**	Unavailable for 170 (4.59%) participants			0.405
0	2,537 (85.85%)	418 (14.15%)	2,955	
1	493 (85.59%)	83 (14.41%)	576	
**Alcohol drinking history**	Unavailable for 76 (2.05%) participants			0.002
0	2,906 (86.87%)	443 (13.13%)	3,375	
1	221 (80.07%)	55 (19.93%)	276	

**Figure 3:**
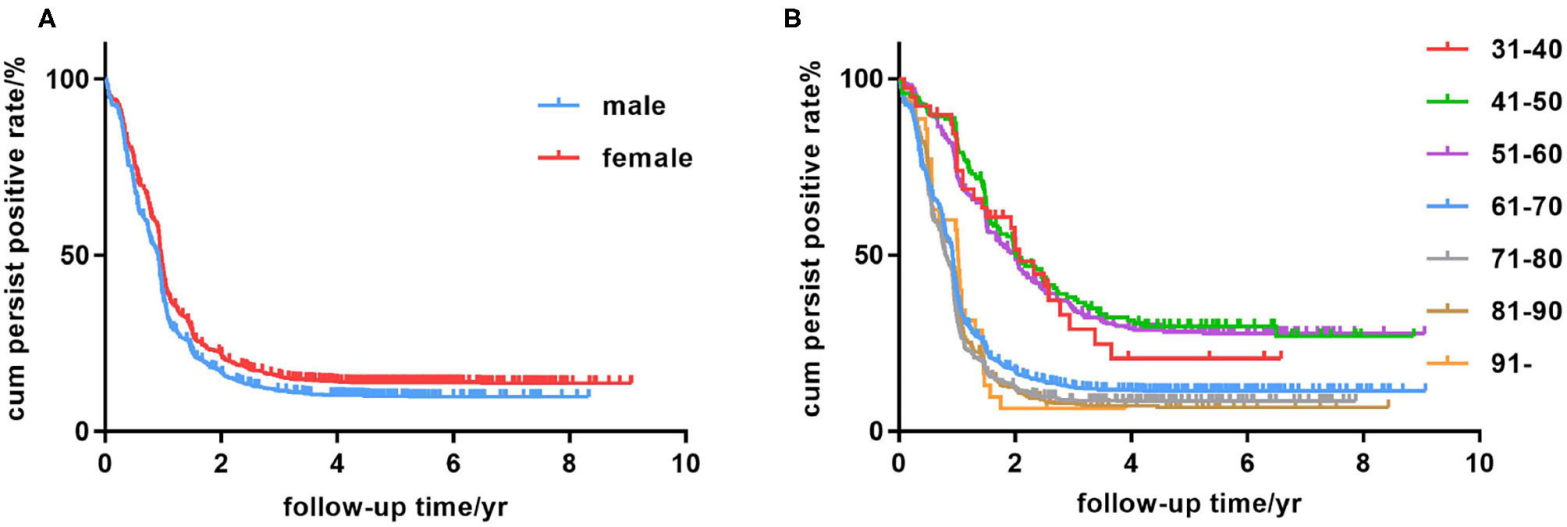
Kaplan-Meier analysis of age group, sex, education level and medical insurance condition. **(A)** Persistent HBsAg positive rate in the male and female groups. **(B)** Persistent HBsAg positive rate in the 31- to 40-, 41- to 50-, 51- to 60-, 61- to 70-, 71- to 80-, 81- to 90-year, and 91-year-and-above age groups.

Kaplan-Meier survival curves illustrated that females were more vulnerable to persistent positive HBsAg than males (Figure 3A). Among various age groups (every 10 years was divided into a group), we observed the younger (21–30-, 31–40-, 41–50- and 51–60 year) groups were more likely to be persistently HBsAg positive than the elderly (61 years and above) groups in a univariate Cox regression (Figure 3B and Table 2). The univariate Cox regression study also demonstrated that female gender, alcohol drinking history and Hepatitis B Family history were potential risk factors for persistent HBsAg positivity. Smoking history don't seem to have a significant impact on the spontaneous clearance rate.

In the following multivariate Cox regression analysis, female gender, middle-age, alcohol drinking history and Hepatitis B Family history were still statistically significant predictors of persistent infection (Table 3). People of female gender were found to have a slightly higher risk for persistently HBsAg positivity with a multivariate-adjusted rate ratio of 1.31 (95% CI, 1.02–1.68). Furthermore, the age stratification analysis found that middle-age populations, especially in the 31- to 40-, 41- to 50-, and 51- to 60 year groups, were prone to being persistently HBsAg positive with hazard ratios of 2.77 (95% CI, 1.57–4.88), 2.76 (95% CI, 1.83–4.17), and 2.63 (95% CI, 1.79–3.86), respectively. Individuals with a history of drinking process a relatively higher risk (hazard ratios of 1.56) contrast to the no-drinking group. Family history also affected the spontaneous clearance rate distinctly. Person with a family history of hepatitis B have approximately twice the risk of chronic infection compared with people from normal family.

**Table 3:** Multi-variate analysis of HBsAg persist positive rate with 95% CI for each predictor.

	**aHR**	**95% CI-low**	**95% CI-high**	***p***
**Gender**				0.033
Male	Reference			
Female	1.31	1.02	1.68	
**Age group**			<0.001	
91	Reference			
21–30	1.55	0.65	3.73	0.327
31–40	2.77	1.57	4.88	<0.001
41–50	2.76	1.83	4.17	<0.001
51–60	2.63	1.79	3.86	<0.001
61–70	1.14	0.8	1.64	0.468
71–80	1	0.69	1.43	0.988
81–90	1.03	0.71	1.49	0.885
**Alcohol drinking history**	1.56	1.06	2.29	0.025
**Hepatitis B Family history**	2.28	1.71	3.04	<0.001

## Discussion

Our study focused on the fluctuation of HBsAg in a large-scale community susceptible population with >140 thousand residents. China government started the national infant hepatitis B vaccination project in 1992 and have not carried out any national adult hepatitis B vaccination policy. So no adult members in this cohort have HBV vaccination history. The annual HBV infection rate was 571.38 per 100,000 person-years, and considering the HBsAg seroclearance rate (86.46%), these results approximate the incidence of hepatitis B (68.58–81.54 per 100,000 person-years) during 2010–2016 from the Chinese public health science data center (19). Still the practical HBV infection rate in this community cohort may be underestimated and be higher than 571.38 per 100,000 person-years on account of some research limitations. For the HBsAg screening interval is 12 months in our research, new-infected people who experienced spontaneous seroclearance within 1 year may be omitted in annual screening. Data reported from national public health organizations were also doomed to underestimate the actual number of cases for the same reason, which was obtained by small-scale a cross-sectional survey (19).

The cohort members of this study are mainly susceptible adult. So the transmission routes could only be unsafe sexual behavior, blood contact and syringe-sharing of drug-addict. Considering the good performance of local government in blood products safety controlling and drug-fighting, most new infections in this study should be sexually transmitted and accidentally blood contact (20). Our survey showed that the incidence rate of new hepatitis B infection for males was significantly higher than that for females (684.88 vs. 536.68 per 100,000 person-years, *p* < 0.001), and 31–40, 41–50, 51–60 age groups had lower new infection rate than younger and elder age groups (Table 1). The phenomenon that males were more likely to have been infected with HBV than females reported by many previous studies could mainly be explained by behavioral differences (11, 21–27). In this research it may relate to the relatively conservative ideology in this county. Female and middle-aged male bearing stable marital status tend to have few extramarital unsafe sexual behavior and accidental blood contact in conservative ideological county, leading to few chance of infection. In this study we found a relatively high rate of new infection in older people. The aging people of this cohort should be speculated to experience more unsafe sex and blood exposure than other age group, contrary to traditional ideas. So it is an unfortunate misunderstanding of our culture that older people are asexual. In this fixed cohort in open community, susceptible people who have common unsafe sexual behavior roughly maintained at a fixed ratio. They were estimated to be all infected (approximately 5 percent of this cohort) in no-intervention state within 10 years (Figure 2). This could also explain the decreasing tendency of annual new infection rate (Supplementary Table 1). At the same time the HBsAg prevalence in young people who have been vaccinated is <1.0% (12). This showed the importance and necessity of vaccine intervention in adult people in a hepatitis B high burden country without adult vaccination coverage.

We also found that 86.46% of patients were observed to have HBsAg seroclearance, furthermore females and individuals between 31 and 60 years were inclined to develop chronicity. This finding is consistent with a recent Chinese community-based study indicating 8.50% chronicity (28). While androgen was widely recognized as a poor prognostic marker for the chronic patient (29–31), in the early stage of infection, serum testosterone may protect the male from chronic hepatitis B infection on account of higher SRD5A2 enzyme activity (32). This may explain the higher risk of persistent infection in female. Seroclearance rates of young and middle age groups in our study approach the result of some previous publications (24, 33, 34). The result that young people under 30 had slightly higher seroclearance rate than middle age group may attribute to their better immuno-competence. However it's really a puzzled phenomenon that age group elder than 60 have distinctly high spontaneous seroclearance rate (Table 2). In theory aging is speculated as negative influencing factor for spontaneous HBsAg seroclearance because aged people usually have lower sex hormone level and immunity. Some researches revealed that aged people have roughly similar or lower spontaneous seroclearance rate contrast to the middle-age group (24, 35). However large-scale real world data of spontaneous HBsAg seroclearance in aging people were few in previous publications. Some other studies appealed opposite findings. For instance a Hong Kong study of 4,568 cohort members and a research of 148 cohort members in Netherlands also found middle-age was associated with developing chronic infection and low HBsAg seroclearance rate compared with aging people (25, 26). The confusing result has not been explained clearly and could partly be due to the increasingly complex innate and adaptive immune responses in elder people (27). High interferon level associated with high viral load in aged individuals may promote the clearance of virus in the early stage (27, 36). Another explanation may be more occult infection occurred in aging people. Occult hepatitis B infection is defined by the persistence of viral genome in the liver in individuals who are tested negative for HBsAg (37). The prevalence data are quite difficult to obtain. In this research the occult infection cannot be simply detected by HBsAg screening. A series of studies found that occult hepatitis B infection prevalence seems to be higher among individuals at high risk of HBV infection and relatively weak baseline liver function status (37–39). So the aging group of our research are more susceptible to occult infection. In spite of the high vanishment rate of HBsAg, resident aging people should receive more liver function monitoring.

We also observed the influence of alcohol drinking history, smoking history and hepatitis family history. It has been reported that alcohol drinking could increase susceptibility to HBV infection and has negative impact at the onset of chronic infection (40). Our research revealed a obviously harmful effect of drinking in new infection individual. They process 1.56 times risk of persistent infection contrast to no-drinking people. Prohibition is a useful suggestion to suppress chronic infections for these susceptible adult community population. Smoking could affect NK cell related antiviral immunity and promote HBV infection progression (41). In this study we found a tiny adverse impact of smoking with no significant statistic difference (Table 2). Smoking may not have a major impact on the early stages of infection. Nonetheless cigarette-controlling is still recommended with other hepatitis health intervention considering the chronic damage of smoking leading to liver dysfunction, cirrhosis and hepatocellular carcinoma (41). People who have hepatitis family history may have more chance of virus exposure and carry more susceptibility genes. A series of SNPs or haplotypes in cytokines, MHC class II and a number of chemokines genes have been found to relate with HBV infection and clearance (42). Our statistics showed that possessing Hepatitis B family history definitely reduced spontaneous clearance. Susceptibility gene screening may be costly in hepatitis B high endemic countries. But appropriate health education could benefit hepatitis families by controlling virus exposure and practicing regular hepatitis screening. In summary more attention should be paid to these people who possess risk factors of chronic infection such as female, middle age, alcohol drinking, and hepatitis B family history (Table 3).

Previous studies proposed that both viral and host factors were determinants of seroclearance; however, this proposal has still not been completely elucidated (4). Infants were believed to have 80–90% chronicity (7, 43–46), and 23–46% of children ≤ 6 years old were chronic carriers of HBsAg in a Taiwanese/Chinese population (47–49). Immune competent adults were the least likely to develop chronic infection with an infection rate of 5–10% (4–6, 28, 50). HBV genotype C2 is more prone to chronic progression than B2 (28).

In conclusion, the results of this study have offered a large-scale real world data of HBV new infection and spontaneous seroclearance in vulnerable adult community people. In this community cohort, the overall infection rate and spontaneous clearance incidence are ~5 and 86%, respectively. Considering the relatively high new infection incidence rate, adult hepatitis B vaccination policy should be considered in hepatitis B high-burden countries. The incidence rate for males was significantly higher than that for females. Female gender, middle-age, alcohol drinking and hepatitis family history were predictors for persistent positive HBsAg status. Continuous follow-up for individuals with the above risk factors are warranted.

A few limitations of this research should be mentioned. Most HBsAg seroclearance cases in our study occurred in the first 2 years, although natural seroclearance of HBV in acute infection is defined as HBsAg clearance in <6 months. It is apparently some HBsAg seroclearance cases have not been monitored. Thus, statistical analysis and study design methods determined that the HBsAg seroclearance interval time should be ~1 year, ideally considering an arbitrarily defined midpoint screening and a 1 year screening. For example, with “negative-positive-negative” consecutive screening results, the time of new hepatitis B infection would be the midpoint of the first and second screenings, and the time of HBsAg seroclearance would be the midpoint of the second and third screenings, making the HBsAg seroclearance interval time exactly 1 year. However, in practice, the time interval between screenings would fluctuate, resulting in HBsAg seroclearance interval times ranging from <1 to 2 years or more. In our large-scale screening we only tested HBsAg as symbol of new infection. This would have omitted a series of occult infection cases. Moreover, elder cohort members were more than young people due to the better compliance. Imbalance of age distribution may lead to bias of total new infection rate and seroclearance rate.

## Data Availability Statement

The raw data supporting the conclusions of this article will be made available by the authors, without undue reservation.

## Author Contributions

LL, KX, and HH designed the study. HH, YS, MH, and YZ collected the data. HH and YS analyzed the data and interpreted the results. HH, YS, and MH wrote the manuscript. LL and KX revised the manuscript from the preliminary draft to submission. LL supervised the whole study. All authors contributed to the article and approved the submitted version.

## Funding

This research was supported by the National Science and Technology Major Project of China (2017ZX10105001).

## Conflict of Interest

The authors declare that the research was conducted in the absence of any commercial or financial relationships that could be construed as a potential conflict of interest.

## Publisher's Note

All claims expressed in this article are solely those of the authors and do not necessarily represent those of their affiliated organizations, or those of the publisher, the editors and the reviewers. Any product that may be evaluated in this article, or claim that may be made by its manufacturer, is not guaranteed or endorsed by the publisher.

## Abbreviations

HBV: hepatitis B virus
HBsAg: hepatitis B virus surface antigen
anti-HBs antibodies: hepatitis B virus surface antibody
WHO: World Health Organization
HR: hazard ratio.
