# Supplementary material for: Incidence and Influencing Factors of New Hepatitis B Infections and Spontaneous Clearance: A Large-Scale, Community-Based Study in China
Source: Front Med (Lausanne). 2021 Nov 18;8:717667. doi: 10.3389/fmed.2021.717667 (PMC8637118; doi:10.3389/fmed.2021.717667)
Supplement: Supplementary file 1 [file Table_1.DOC]

**Supplementary material**

**Supplementary Table 1**. Cumulative incidence and annual incidence rates of HBsAg positive conversion

| Year | 1 | 2 | 3 | 4 | 5 | 6 | 7 | 8 | 9 | 10 |
| --- | --- | --- | --- | --- | --- | --- | --- | --- | --- | --- |
| Cumulative incidence rate | 0.007 | 0.011 | 0.018 | 0.023 | 0.028 | 0.035 | 0.041 | 0.044 | 0.045 | 0.047 |
| SE | <0.001 | <0.001 | <0.001 | <0.001 | 0.001 | 0.001 | 0.001 | 0.001 | 0.001 | 0.002 |
| Annual incidence rate (per 100000) | 670.58 | 445.27 | 603.31 | 474.98 | 289.46 | 300.48 | 168.12 | 57.65 | 1.36 | 0.68 |
| Trend p | <0.001 |  |  |  |  |  |  |  |  |  |

**Supplementary Table 2**. Cumulative incidence and annual incidence rates of HBsAg seroclearance

| Year | 1 | 2 | 3 | 4 | 5 | Final |
| --- | --- | --- | --- | --- | --- | --- |
| Cumulative incidence rate | 0.573 | 0.802 | 0.861 | 0.861 | 0.861 | 0.861 |
| SE | 0.008 | 0.007 | 0.006 | 0.006 | 0.006 | 0.006 |
| Annual incidence rate | 0.462 | 0.526 | 0.265 | 0.087 | 0.002 | / |
| Trend p (1-2 yr vs. 3 yr and later) <0.001 | | | | | | |

**Author Permissions**

**
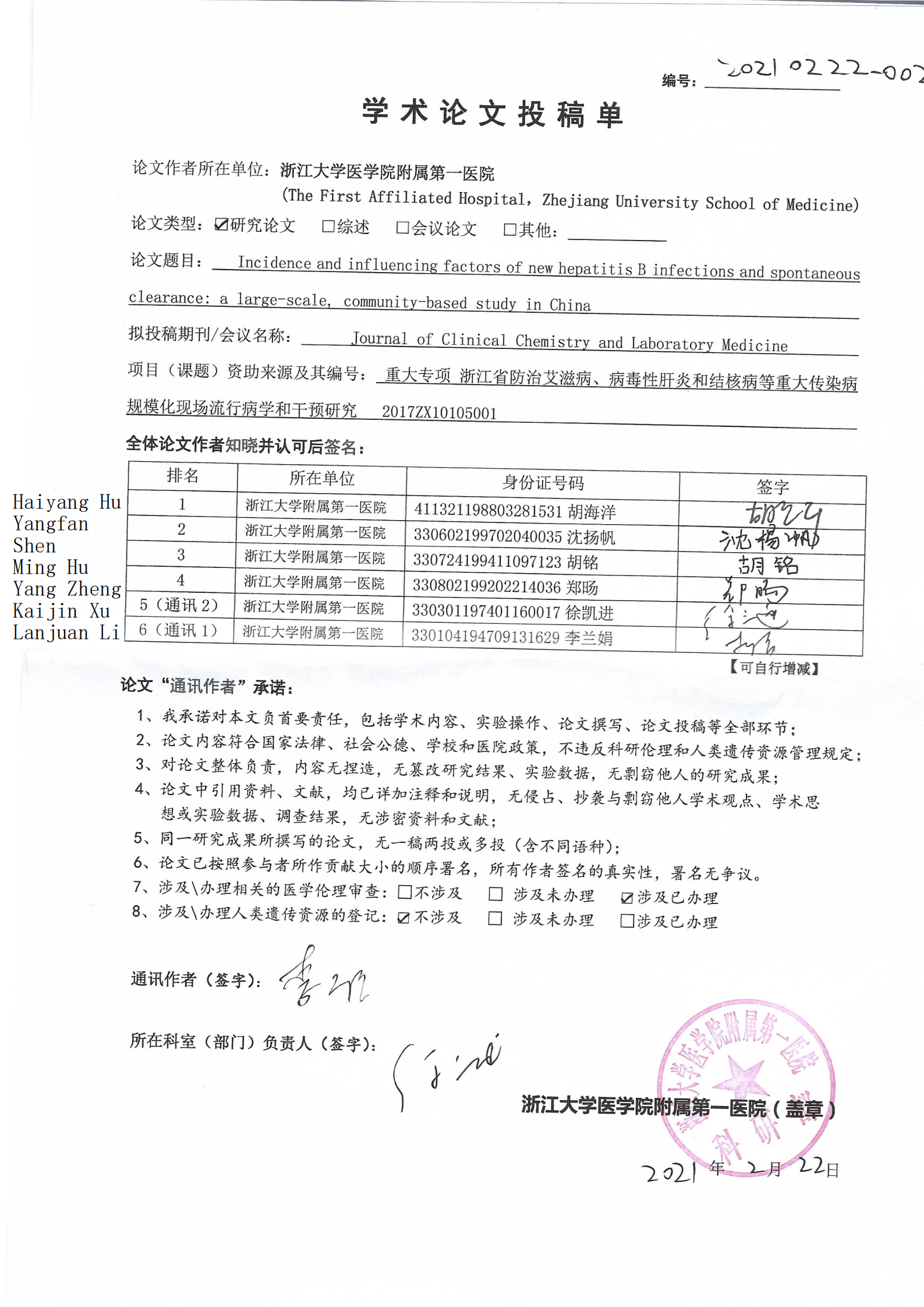
**

**Ethical Approval**

**
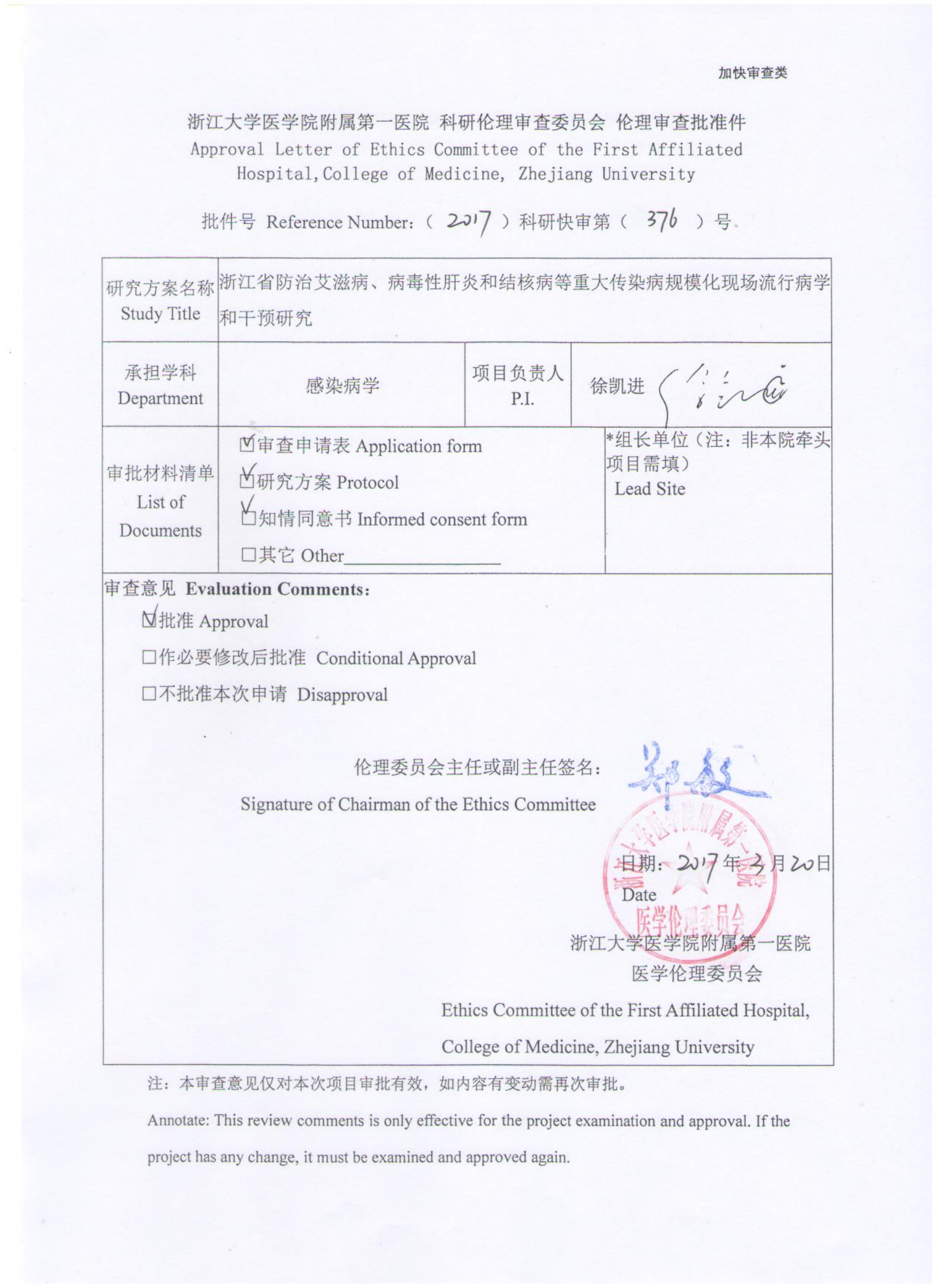

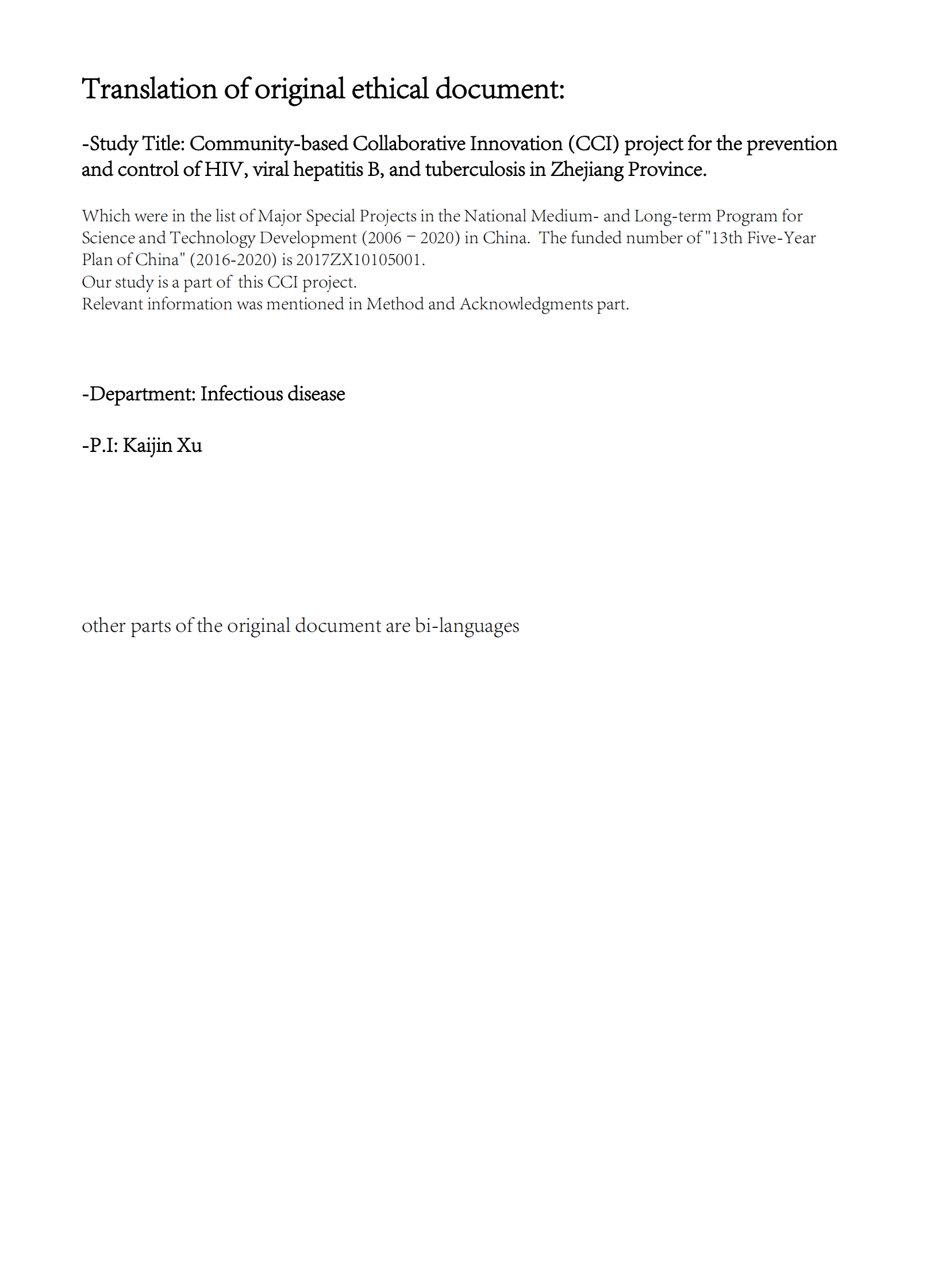
**

**Inform Consent**

**
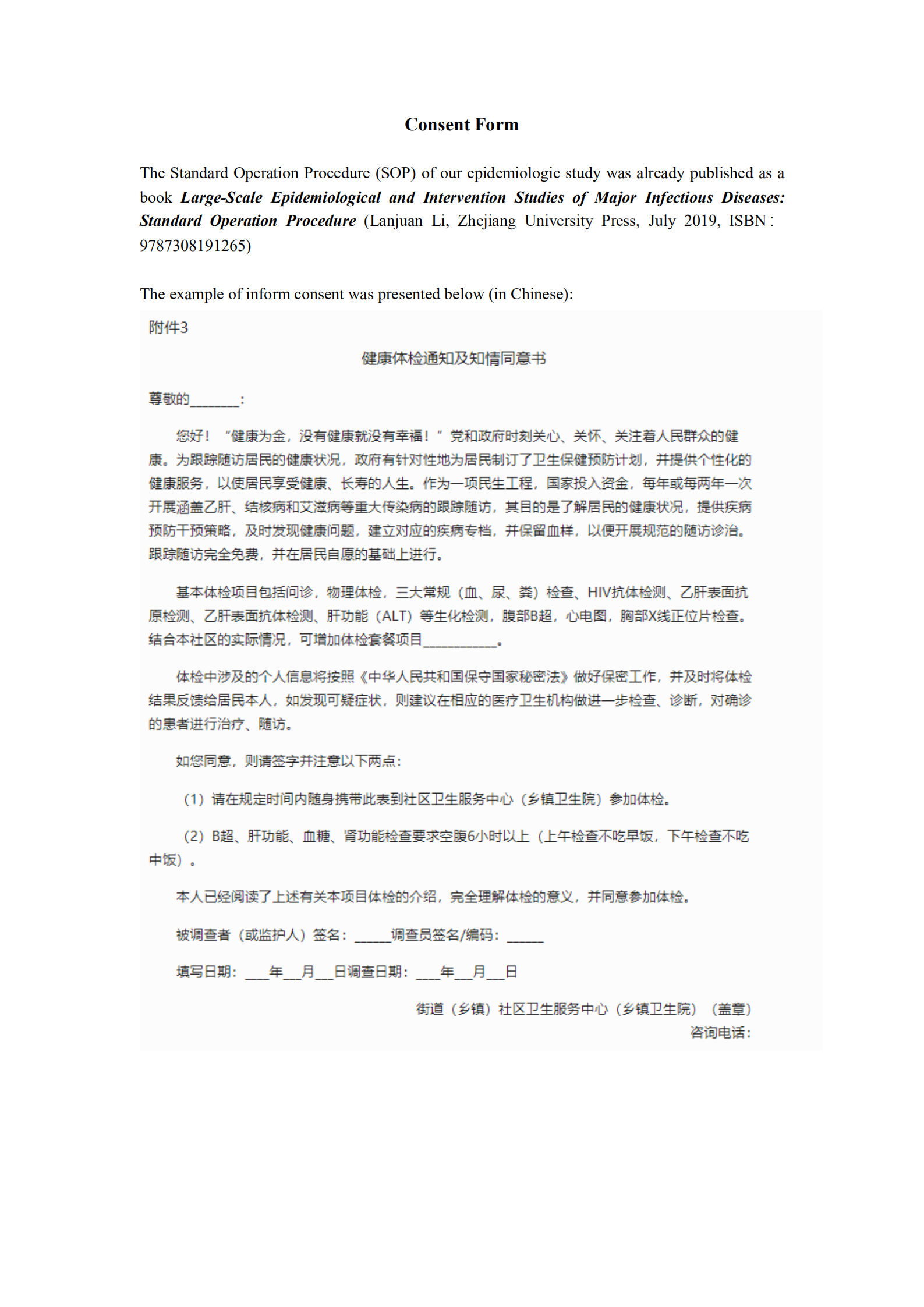

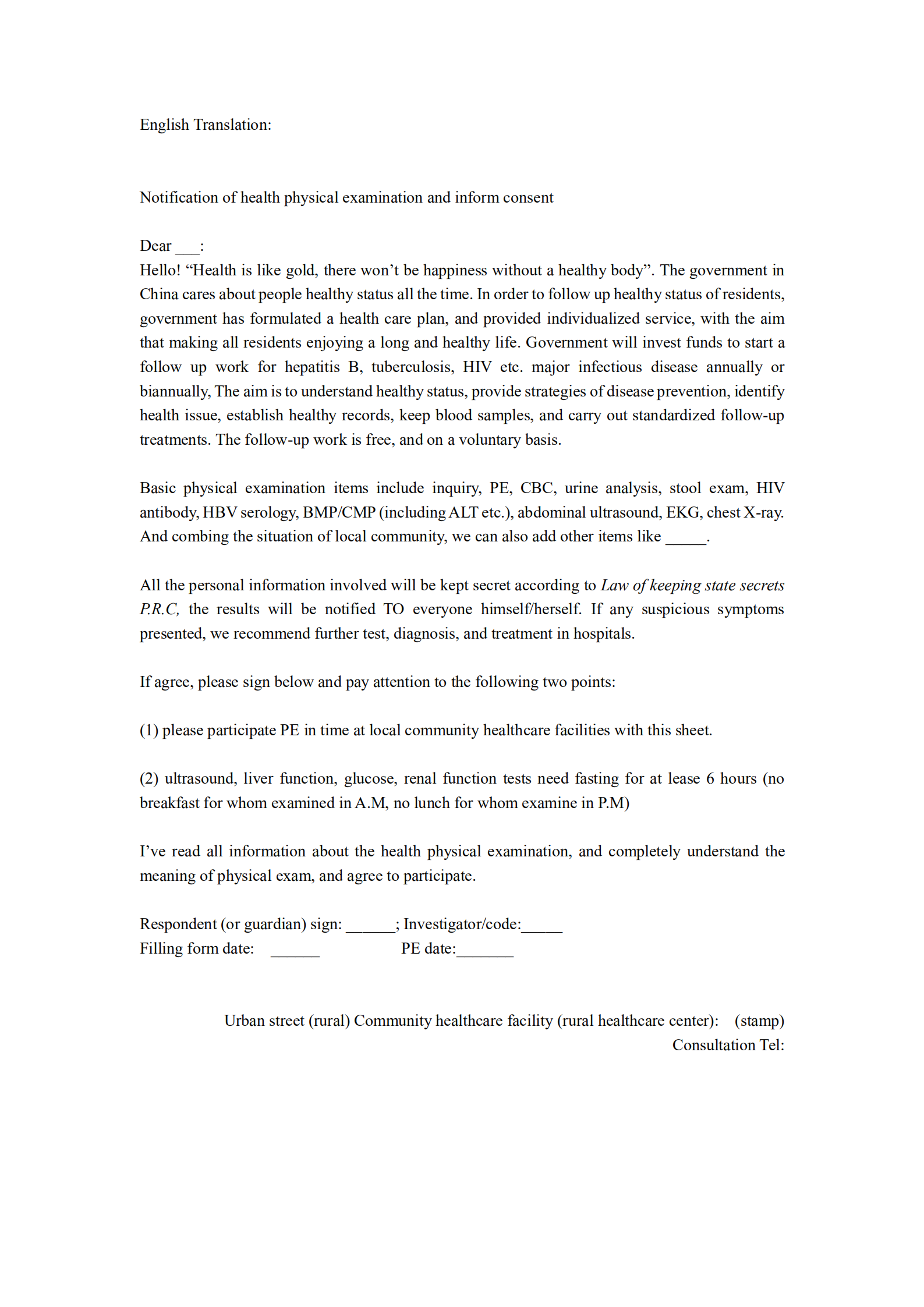
**
